# Social acceptance and population confidence in telehealth in Quebec

**DOI:** 10.1186/s12913-015-0727-1

**Published:** 2015-02-21

**Authors:** Thomas G Poder, Christian A Bellemare, Suzanne K Bédard, Renald Lemieux

**Affiliations:** UETMIS and CRCHUS, CHUS Hôtel-Dieu, 580 rue Bowen Sud, J1G 2E8 Sherbrooke, QC Canada; DSQ, MSSS, Montréal, QC Canada

**Keywords:** Telehealth, Social acceptance, Confidence, Population, Quebec

## Abstract

**Background:**

Access to healthcare in remote areas is difficult and telehealth could be a promising avenue if accepted by the population. The aim of this study is to assess social acceptance and population confidence in telehealth in the Province of Quebec.

**Methods:**

We conducted a survey using a questionnaire assessing the social acceptance of and confidence level in telehealth. Two strategies were used: 1) paper questionnaires were sent to two hospitals in Quebec; and 2) online questionnaires were randomly sent by a firm specialized in online survey to a representative sample of the population of the Province of Quebec. Respondents were all residents of the Province of Quebec and 18 years and older. Questions were scored with a four-level Likert scale.

**Results:**

A total of 1816 questionnaires were analyzed (229 written and 1,587 online questionnaires). The socio-demographic variables in our samples, especially the online questionnaires, were fairly representative of Quebec’s population. Overall, social acceptance scored at 77.71% and confidence level at 65.76%. Both scores were higher in the case of treatment (3 scenarios were proposed) vs. diagnosis (p < 0.05). No difference was found when respondents were asked to respond for themselves and for a member of their family, which demonstrates a true interest in telehealth in Quebec. In addition, we found a significant difference (p < 0.05) between written and online questionnaires regarding social acceptance (80.75% vs. 77.33%) and confidence level (74.84% vs. 64.55%). These differences may be due to social desirability or avidity bias in the written questionnaires.

**Conclusions:**

Our results suggest that the population in Quebec encourages the development of telehealth for real time diagnosis and long distance treatment for regions deprived of healthcare professionals.

**Electronic supplementary material:**

The online version of this article (doi:10.1186/s12913-015-0727-1) contains supplementary material, which is available to authorized users.

## Background

A fundamental principle of Quebec’s health care system is equal access to healthcare services. Indeed, the funding of this system is based on general taxation. This orientation spreads risk equitably throughout the society. Unfortunately, this goal is not always achievable in practice, especially for patients living in rural areas where the lack of specialized services is generally an issue. For example, some regions are deprived of a neurosurgeon or cardiologist. Moreover, the exposure and practices of the medical personnel are not the same in rural as in urban hospitals. The number of cases in need of a specialized care per year and the proximity of a university healthcare center can explain this situation. The *de facto* ability to provide such services to all requires, 1) the development of a large Quebec medical clinic network and a strong organization providing patient care in the community, or 2) patient transportation to urban centers to receive the required care. The size of Quebec’s territory and its low population density make the first solution difficult to operate. The second solution, currently in use, is very expensive and highly limited in cases such as an emergency situation when the patient is in danger of losing his life. This situation is very challenging because the patient must be treated locally by a medical team. An alternative solution to this problem is the development of telehealth care to provide local health services assisted by specialized health care professionals.

Increase in equal access to medical services in a number of specialties, such as psychiatry, geriatrics, rheumatology, and dermatology, is a factor affecting the success of telehealth care [1-3]. For example, in the survey conducted by Davis et al. [1], patients were asked about their options if no telehealth consultation was available. Of the 53 patients sollicited, 13 said that they would not have been bothered if they did not have any consultation and 37 said that they would have to travel. Some studies have also demonstrated cost savings [4,5] and greater or equal quality of care in telehealth settings [6]. However, the systematic review of Wade et al. [5] concluded that it was only cost-effective for home care and access to on-call hospital specialists and sometimes for rural service delivery, whereas it was not for local delivery of services between hospitals and primary care. The results are also encouraging for more acute care specialties, such as trauma and wound care [7-10]. Boulanger et al. [7] present in their study 22 outpatient follow up for trauma through telemedicine with a high degree of patient satisfaction and a significant reduction of travel distances. From their side, Cabrera et al. [8] estimated a €6,030 cost savings per 100 patients when transmitting electrocardiograms and images from ambulances to the healthcare centers. Lafiti et al. [10] showed, in a 13 months pilot study about tele-trauma and telepresence for injured patients, that five out of 21 patients were managed through telemedicine without being transferred to a Level 1 trauma center. Another point to consider in developing telehealth services is to know the differences between rural and urban hospitals using telemedicine. Ward et al. [11] addressed this topic in their study. From 4,727 U.S. hospitals registered in the 2013 Health Information and Management System Society, 66% and 68% of rural and urban hospitals had no telehealth services, respectively. Among hospitals with operational telehealth services, urban hospitals had more multiple telehealth services than rural hospitals (42.1% vs 35.2%). Moreover, urban hospitals implemented more cardiology, neurorogy and obstetrics telehealth services than rural hospitals. In contrast, rural hospitals developed more radiology and emergency telehealth programs than urban hospitals.

Telehealth can be generally defined as a means of sharing health information and health care services through interactive audio/video communication and computer technology. It improves the ability of health professionals to provide consistent and quality health care services regardless of the geographic location, and it is a very promising option to compensate for the shortage of specialized healthcare professionals and to improve quality of services, especially in rural regions. More formally, the American Telemedicine Association describes telemedicine as *the use of medical information exchanged from one site to another via electronic communications to improve a patient’s clinical health status* [12]*.* More contextualized with this paper, Canada Health Infoway define telehealth as *the use of communications and information technology to deliver health care services over large and small distances, including remote and rural areas* [13].

Although a technology or service used in a telehealth care program may meet the requirement to export medical expertise in remote regions and may prove to be very effective, the technology can pose a very significant problem during use or intended use [14]. These problems largely refer to the notion of acceptance of telehealth, which currently remains widely understudied. Indeed, most studies on the use of telehealth are limited to aspects such as ease and frequency of use, advantages and costs, format and the relevance and accuracy of the information delivered. There is little research on the acceptance of telehealth and studies focus more on individual acceptance, including ergonomics and user satisfaction [15], rather than on social acceptance [16,17]. A study of the use of telehealth must consider three dimensions: utility, usability and social acceptance [14]. The measurement of these three dimensions may prove useful in predicting how the technology will be utilized.

Some studies assessed the perception of patients toward the use of telehealth. For example, Edwards et al. [18] were interested about patient perception in chronic diseases follow up using telehealth. Of the 3,329 questionnaires sent to patients with depression and cardiovascular disease, 44.40% completed it. They found a moderate interest in the use of phone-based (60.01%) and email/Internet-based (57.26%) telehealth services. Their results are less encouraging regarding social media with a 16.99% interest. In the study of Vodicka et al. [19], privacy was considered. In this dimension, they looked at patient’s attitude towards the electronic access to their medical records. Out of the 3,874 respondents, they found 32.91% of patients who reported concerns about privacy. These results show the importance of addressing patient’s perception before introducing electronic technologies in healthcare. Indeed, Beck [20] states that the social acceptance of a project is directly related to the perceived threats to individual lives or quality of life. Social acceptance thus corresponds with the early acceptance of short and long term risks inherent to a project or a situation. When a risk is considered acceptable, a community can accept consequences and damages in terms of probability of occurrence. Given the social attitudes, constraints, and standards in each society, social acceptance for a specific technology exists if a community resorts to its uses.

A study assessing the potential user social acceptance of telehealth in the context of Quebec’s health care system is considered essential to successfully implement a trauma care telehealth project, which installs audio and video technologies in an emergency room. This research aims to assess social acceptance and population confidence in telehealth in the Province of Quebec as a prerequisite to such a project.

### Telehealth trauma care in emergency room

Four health university networks in the Province of Quebec developed health care programs with financial support from Canada Health Infoway. One network developed a telewound care management program that included over 65 territorial service points in 2011. Teletraumatology is another program envisioned by the same university network (Réseau universitaire intégré de santé de l’université de Sherbrooke).

Identified by a pilot study conducted at the Centre hospitalier universitaire de Sherbrooke (CHUS), teletraumatology is a solution to assist local clinicians in performing stabilization invasive procedures on polytraumatized patients to compensate for the shortage of emergency specialists and maintain the quality of medical care in remote regions. Because no robot-based systems for trauma medicine are available in the market, a robotized camera system has been developed by the Centre hospitalier universitaire de Sherbrooke (CHUS), the Faculty of Medicine and Health Sciences (FMHS) and the Faculty of Engineering of the Université de Sherbrooke.

The team developed a system [21] that uses two mobile cameras ceiling-mounted at the end of an Articulated Robot Arm. The PAN (horizontal rotation), TILT (vertical rotation) and ZOOM (magnification factor) capabilities of the cameras and their positioning above the stretcher are controlled remotely. This system provides real-time communication between a remote trauma surgeon and an on-site emergency physician to allow a trauma surgeon to remotely assist a local clinician as if the surgeon were with the clinician in the emergency room.

Our assessment of the social acceptance and population confidence in telehealth, including specific section in the field of teletrauma, was conducted prior to a clinical assessment of the teletraumatology system.

## Methods

We surveyed in 2009–2010 the population of Quebec on telehealth social acceptance and confidence through a questionnaire (see Additional file 1) administered in French in two ways: 1) a paper questionnaire was handed out in two hospital waiting rooms in the Eastern Township region (CHUS and Brome-Missisquoi-Perkins (BMP)); and 2) an online questionnaire was sent out to the general population of Quebec. The distribution of the paper questionnaire was conducted by recruiting subjects at their convenience in both emergency rooms (ER) and outpatient clinics waiting areas in each hospital. For the online questionnaire, we asked an online survey company (Survey Sampling International) to conduct a random selection of its panelists and send requests for study participation.

Our study was approved by our University Hospital Research Center Ethics committee. Inclusion criteria were 1) Quebec residents and 2) 18 years and older. In the hospitals, patients were approached by a research assistant who explained the purpose of the research and after they manifested their intent to participate, they were given out the survey accompanied by an explanatory letter. The participants filled out the survey either before or while waiting for their care in the doctor’s office.

The questionnaire was divided into three sections. The first section included socio-demographic data to define respondent profiles. The second section briefly described telehealth and explained the reasons driving its development in the Province of Quebec. Finally, the third section included a total of 16 questions divided into four series of four questions on telehealth social acceptance and confidence. Each series consisted of a question on the degree of agreement with using telehealth to access healthcare services (based on a Likert scale with four levels of agreement defined as *total, moderate, weak or not at all*), immediately followed by a question about the degree of confidence in telehealth (assessed using a 21-step percentage points score from 0 to 100%, i.e. divided by 21 to have a graduation every 5 points). Respondents answered each question twice: once for itself and once for a member of its family. The four series of questions considered themes in the following order: 1) real-time remote assistance to establish a diagnosis or a treatment plan; 2) real-time remote assistance of a physician for executing specialized treatments; 3) real-time remote assistance of a healthcare professional (nurse, physiotherapist, etc.) for executing specialized treatments; and 4) real-time remote assistance of an ER physician for performing stabilization invasive procedures on polytraumatized patients in the emergency room.

The study questionnaire was developed from a search of the literature on social acceptance of telehealth to consider the various dimensions addressed in this study. This questionnaire was adapted to the linguistic context in Quebec, and hospital research professionals validated each survey statement for relevance, consistency, accuracy, and lack of ambiguity. Pre-tests were conducted with secretaries and outpatients in our hospital (N = 7). The pre-test respondents indicated that the questionnaire would be comprehensible for the general population and identified the time in minutes required to answer all questions.

### Statistical data analysis

The degree of agreement total score for each respondent is equal to the sum of individual scores attached to all 8 questions answered. Total score varies from 0 to 24 because each question had a score corresponding to the Likert level chosen (*total (3), moderate (2), weak (1)* or *not at all (0))*. The degree of confidence mean score for each respondent is the mean of the percentage scores related to all 8 questions answered. The mean score varies from 0 to 100%.

Student’s *t*-test was used to compare differences between two groups of variables represented by real numbers, such as *age, year of education*, *income, social acceptance* and *confidence scores*. Pearson’s chi-squared was used to compare differences between two groups of variables represented by percentages, such as *male, employment, single* and *children*. Groups comparison were sample vs. general population (*Total survey* vs. *Stat. Québec*), electronic vs. paper form questionnaire (*Internet* vs. *Written*), university vs. regional hospital (*CHUS* vs. *BMP*), and type of waiting area (*Emergency room* vs. *Outpatient clinic*). Multiple linear regressions were used to model the relationships between independent variables and dependent variables. Statistical tests were conducted with STATA 8.0 and Rproject 2.9.2 on Microsoft Windows XP 2005. Statistical significance was set at p < 0.05.

## Results

This survey allowed us to collect 1,816 questionnaires with at least one completed question about social acceptance and population confidence, out of which 1,738 were 100% complete. A total of 6,000 participants were solicited, resulting in a response rate of 30.3%. Out of the 1,816 questionnaires, 1,587 were online questionnaires, and 229 were written questionnaires from a medical center (Centre hospitalier universitaire de Sherbrooke (CHUS) [N=202] and Brome-Missisquoi-Perkins Hospital (BMP) [N=27]). Out of these 229 questionnaires, 102 were completed in emergency rooms, 119 were completed in outpatient clinics (OC) and 8 were completed without respondent specification of ER or OC.

Statistics Canada (2008, 2010) provided provincial statistics for all variables except employment rate and the number of years of education, which were provided by the Institut de la statistique du Québec (2008, 2010). Variable units differ; for instance, the age variable is in years, the male variable is a percent, the education variable is the number of schooling years beginning from age 6, the employment variable is the percent of respondents with a part time job or a full time job, the gross income variable is in Canadian dollars, the single variable is the percent of respondents neither married nor with a partner, and the children’s variable is the percent of respondents with children.

Table 1 show that respondents who answered via Internet were mature adults, with a nearly equal proportion of males and females. The respondents finished high school, and more than half were still working. The average annual income for Internet respondents was just over $36,000. Most of respondents lived with someone and had children. However, respondents who answered the paper questionnaire were also mature adults, but mostly female. These respondents also finished high school and more than half of them were still working. The average annual income for paper respondents was just over $35,000. These respondents also lived with someone and had children. Further stratification of this latter group shows that a larger proportion of female answered the paper questionnaire at the regional hospital BMP rather than at the university hospital CHUS. BMP respondents had an income that was slightly lower than CHUS respondents. The respondents who visited the outpatient clinics were generally about ten years older than the respondents who visited the emergency room.

**Table 1:** **Socio-demographic respondent characteristics (N = 1,816)**

	**Age (years)**	**Male (%)**	**Education (# years)**	**Employment (%)**	**Income ($)**	**Single (%)**	**Children (%)**
Stat. Québec^a^	**47.9**	49.0	**10.7**	**59.7**	35,400	38.0	--
Total survey	**46.5 ± 14.9**	51.0	**11.3 ± 2.2**	**56.4**	35,985 ± 24,124	36.4	67.5
(N = 1,816)	(N = 1,809)	(N = 1,814)	(N = 1,813)	(N = 1,811)	(N = 1,781)	(N = 1,796)	(N = 1,811)
Internet	46.6 ± 14.52	**52.7**	**11.2 ± 2.1**	**55.4**	36,086 ± 24,254	36.6	**66.5**
(N = 1,587)	(N = 1,587)	(N = 1,587)	(N = 1,587)	(N = 1,587)	(N = 1,586)	(N = 1,573)	(N = 1,587)
Written	45.6 ± 17.4	**39.2**	**11.9 ± 2.8**	**62.9**	35,167 ± 23,094	35.0	**74.1**
(N = 229)	(N = 222)	(N = 227)	(N = 226)	(N = 224)	(N = 195)	(N = 223)	(N = 224)
CHUS	45.7 ± 17.5	40.5	11.9 ± 2.9	61.6	35,485 ± 23,739	**37.8**	74.1
(N = 202)	(N = 195)	(N = 200)	(N = 199)	(N = 198)	(N = 170)	(N = 196)	(N = 197)
BMP	44 ± 17.3	29.6	11.6 ± 2.1	73.1	33,000 ± 18,357	**14.8**	74.1
(N = 27)	(N = 27)	(N = 27)	(N = 27)	(N = 26)	(N = 25)	(N = 27)	(N = 27)
Emergency	**40.1 ± 18.3**	33.7	12.1 ± 2.6	67.0	34,265 ± 23,308	**42.0**	**64.7**
(N = 102)	(N = 100)	(N = 101)	(N = 101)	(N = 100)	(N = 85)	(N = 100)	(N = 102)
Outpatient	**50.5 ± 15.0**	45.4	11.8 ± 3.1	57.3	36,178 ± 23,325	**28.5**	**81.7**
(N = 119)	(N = 116)	(N = 119)	(N = 118)	(N = 117)	(N = 104)	(N = 116)	(N = 115)

^a^Standard deviations are not available for Quebec statistics.

Significant differences are in bold at p < 0.05.

The socio-demographic characteristics of the respondents in our sample are fairly close to the population of Quebec as depicted by the provincial statistics. Statistical analysis with a one-sample Student’s *t*-test showed a significant difference between the two groups regarding variables *age, years of education* and *employment rate*, although these differences are small (1.4 years of age, 0.6 years of education and 3.3 points in employment rate). Such small differences do not indicate that our sample is not representative of the population of Quebec. We believe that these small differences were significant because of our large sample size (N = 1,816). This sample size increases the sample power at 95% confidence interval to a point near 97%, where such small differences become significant. For example, the 95% confidence interval is [45.8, 47.2] for age, [11.2, 11.4] for years of education and [55.2, 57.5] for employment rate. All provincial and national statistical data for these variables is outside these intervals.

A sample selection bias may cause the significant differences between the group “*Online Internet questionnaires*” and the group “*Written questionnaires*” for the following variables, as indicated in Table 1: *male, education, employment* and *children*. Indeed, the very small number of written questionnaires (229) compared to the much larger number of Internet questionnaires (1,587) increases the risk of such a bias. Additionally, the Internet survey recruitment strategy structured the sample like the general population; we asked the survey company to select a sample group composed of 50% males and 50% females and to randomly recruit members of each group.

Similarly, the significant difference observed between the group “*CHUS*” and the group “*BMP*” for the variable *single* may be due to selection bias because a very small number of questionnaires (27) were completed at BMP compared to the 202 questionnaires completed at CHUS. However, we cannot eliminate the possibility that single people are more represented in the *“BMP”* sample than the *“CHUS”* sample.

When comparing the group “*Emergency room*” (ER) with the group “*Outpatient clinic*” (OC), we observe significant differences for the *age, single* and *children* variables. Because the number of respondents was quite similar in both groups (102 *vs.* 119), our first interpretation suggests that the clientele visiting the ER differs from OC clientele, regardless of the hospital visited. Briefly, data show that the OC clientele is older, lived with someone, and had children. If we specifically look at the variable *age*, the OC group is on average 10.4 years older than the ER group (p < 0.001). The difference in age between the OC and ER groups seems to be confirmed by the median age values: 51.7 years for the OC patients *vs.* 35.5 years for the ER patients. Most members of the OC group lived with someone (>70%), and over 80% had children, compared to 64.7% for the ER group (p < 0.005). Those significant differences in group characteristics are consistent with our general observations of the population visiting the ER and the OC.

Tables 2 and 3 detail the telehealth social acceptance scores and confidence percentage levels for consultation with a physician or other healthcare professional, diagnostic purposes or treatment from the same professionals. The frequency distribution of answers is given for each question in Additional file 2.

**Table 2:** **Answers to questions 1 and 2 on social acceptance (mean score [0;3] ± SD) and confidence level (mean score in % ± SD)**

	**Q1a**	**Q1ap (%)**	**Q1b**	**Q1bp (%)**	**Q2a**	**Q2ap (%)**	**Q2b**	**Q2bp (%)**
Total	2.0 ± 1.0	56.4 ± 29.7	2.0 ± 0.9	56.0 ± 29.9	2.4 ± 0.8	67.5 ± 27.6	2.4 ± 0.8	67.2 ± 27.8
Internet	2.0 ± 1.0	54.8 ± 29.6	2.0 ± 0.9	54.4 ± 29.9	2.4 ± 0.8	66.4 ± 27.9	2.4 ± 0.8	65.9 ± 28.2
Written	2.3 ± 0.9	68.5 ± 26.9	2.2 ± 0.9	67.8 ± 27.5	2.4 ± 0.8	75.4 ± 24.4	2.4 ± 0.8	76.8 ± 23.0
CHUS	2.2 ± 0.9	66.5 ± 27.8	2.1 ± 0.9	65.3 ± 28.3	2.4 ± 0.9	73.5 ± 25.3	2.4 ± 0.8	75.0 ± 23.8
BMP	2.7 ± 0.5	83.1 ± 11.2	2.7 ± 0.5	85.4 ± 8.5	2.8 ± 0.4	88.3 ± 10.3	2.8 ± 0.4	89.3 ± 9.9
Emergency	2.3 ± 0.9	69.6 ± 26.6	2.2 ± 0.9	69.1 ± 27.9	2.4 ± 0.8	78.9 ± 22.0	2.5 ± 0.7	80.6 ± 19.9
Outpatient	2.2 ± 0.9	67.7 ± 26.8	2.1 ± 0.9	66.9 ± 26.8	2.4 ± 0.9	72.2 ± 26.0	2.4 ± 0.9	73.4 ± 24.9
Nb. Obs.	1,807	1,806	1,807	1,805	1,780	1,775	1,780	1,777
Interaction	Patient ↔ Physician				Physician ↔ Specialist

**Table 3:** **Answers to questions 3 and 4 on social acceptance (mean score [0;3] ± SD) and confidence level (mean score in % ± SD)**

	**Q3a**	**Q3ap**	**Q3b**	**Q3bp**	**Q4a**	**Q4ap**	**Q4b**	**Q4bp**
Total	2.4 ± 0.8	67.2 ± 27.8	2.4 ± 0.8	66.5 ± 28.0	2.6 ± 0.7	72.6 ± 26.5	2.5 ± 0.7	72.0 ± 26.7
Internet	2.4 ± 0.8	66.1 ± 28.1	2.3 ± 0.8	65.5 ± 28.3	2.6 ± 0.7	71.5 ± 26.9	2.5 ± 0.7	70.9 ± 27.2
Written	2.4 ± 0.8	75.2 ± 24.3	2.4 ± 0.8	74.4 ± 25.0	2.6 ± 0.6	80.9 ± 21.1	2.6 ± 0.7	80.6 ± 21.0
CHUS	2.4 ± 0.8	73.3 ± 25.2	2.4 ± 0.8	72.3 ± 25.9	2.6 ± 0.7	80.0 ± 21.8	2.6 ± 0.7	79.6 ± 21.8
BMP	2.7 ± 0.4	88.5 ± 10.0	2.7 ± 0.4	88.5 ± 9.9	2.7 ± 0.5	87.8 ± 13.2	2.7 ± 0.5	87.6 ± 12.5
Emergency	2.5 ± 0.6	78.2 ± 20.1	2.5 ± 0.6	77.6 ± 21.6	2.6 ± 0.7	82.7 ± 20.2	2.5 ± 0.7	82.0 ± 20.5
Outpatient	2.4 ± 0.9	72.6 ± 26.7	2.3 ± 0.9	71.6 ± 27.1	2.7 ± 0.6	79.5 ± 21.4	2.7 ± 0.7	79.7 ± 21.1
Nb. Obs.	1,769	1,763	1,770	1,764	1,755	1,750	1,753	1,751
Interaction	Healthcare professional ↔ Specialist				ER physician ↔ Specialist			

The methodology section notes that question Q1 focuses on patient telehealth usage to consult a physician for a diagnosis or to plan a treatment. Question Q2 focuses on the family physician using telehealth to consult a specialist and perform a specialized technique to treat a patient. Question Q3 details the same situation as question Q2, but considers other healthcare professionals instead of physicians. Finally, question Q4 focuses on an emergency situation where a traumatology specialist uses telehealth to provide real-time assistance to an ER physician during the execution of specialized techniques to stabilize polytraumatized patients. The respondent answers for oneself (questions Qxa) or for a member of its family (questions Qxb) in all of these situations. All questions ending with a “p” focus on respondent confidence level in using telehealth in the above situations.

Tables 2 and 3 show that the social acceptance score of the overall respondent sample generally increases from 2.0 to 2.6 as the intervention moves from diagnostic (Q1) to treatment (Q2 & Q3) and as the situation becomes more critical (Q4). This finding holds true for all respondents except for those from the regional hospital BMP, where scores are higher and quite stable (between 2.7 and 2.8) in all situations. A similar behavior of confidence scores was observed.

Except for BMP, we found a statistically significant increase (p < 0.001) in the social acceptance and confidence scores when Q1 is compared to Q2, Q3 and Q4. We interpret these findings as the respondents better accepting telehealth usage when a physician or another healthcare professional is assisted by a specialist during a specialized treatment; in contrast, respondents are more reluctant to use telehealth to consult a physician to establish a diagnosis or to plan their treatment. The respondents may prefer a face-to-face consultation in this last situation. However, respondents do not differentiate whether the professional using telehealth is a physician or other healthcare professional; we do not see any significant difference between Q2 and Q3. Finally, levels of acceptance and confidence in using telehealth are the highest when the health condition is more critical (Q4) and requires more urgent interventions from the healthcare professionals, such as stabilization of trauma patients in the emergency room.

For every dimension of social acceptance, expressed by Q1 to Q4, the level of acceptance and confidence do not differ significantly (p = 0.27, p = 0.60, respectively) when the respondent answer for oneself or for a family member. This result seems to indicate that respondents express a high level of empathy toward members of their family and that easily project their own level of acceptance of telehealth usage onto their family members.

To determine the overall social acceptance results, we only kept questionnaires that fully completed all eight questions, for a total of 1,745 for the level of acceptance and 1,742 for the confidence level. The overall level of acceptance is based on a maximum global score of 24 earned by receiving the maximum score of 3 for each question (3 × 8 = 24), where 3 is quoted as *total agreement*, 2 is quoted as *moderate agreement*, 1 is quoted as *little agreement*, and 0 is quoted as *no agreement*. Table 4 shows the level of acceptance as the mean score ± standard deviation. The confidence level is expressed as a percentage from 0% to 100%, where 0% indicates *no confidence* and 100% indicates *total confidence*. Table 4 shows the mean percentage ± standard deviation.

**Table 4:** **Overall results (mean ± SD) of social acceptance and confidence level in telehealth**

	**Social acceptance**		**Confidence level**	
	**Score on 24**	**Nb. Obs.**	**Average percentage**	**Nb. Obs.**
Total	18.65 ± 5.24	1,745	65.76 ± 24.58	1,742
Internet	18.56 ± 5.26	1,538	64.55 ± 24.80	1,538
Written	19.38 ± 5.08	207	74.84 ± 20.82	204
CHUS	19.01 ± 5.23	180	73.08 ± 21.48	178
BMP	21.85 ± 3.00	27	86.90 ± 8.92	26
Emergency	19.56 ± 4.75	93	77.02 ± 19.03	91
Outpatient	19.09 ± 5.42	108	73.04 ± 21.66	107
	**Diff.**	**P-value**	**Diff.**	**P-value**
Internet vs. Written	**0.82**	0.034	**10.29**	0.000
CHUS vs. BMP	**2.85**	0.006	**13.82**	0.001
Emergency vs. Outpatient	−0.47	0.520	−3.98	0.175

Significant difference in bold at p < 0.05.

The significant difference that we observe between the Internet and Written questionnaires in Table 4 may be due to a sample selection bias previously indicated but may also be attributable to a difference in group behavior. Indeed, social desirability bias may exist in the paper questionnaires distributed by an interviewer. Social desirability bias occurs when individuals in the presence of the interviewer provide different responses to appear in a favorable light. This situation may have occurred in paper questionnaires that were administered by interviewers. However, the Internet format may provide more time for reflection because an interviewer is not waiting for a response and may be less subject to social desirability bias [22,23]. An avidity bias may also be present. Avidity bias refers to the notion that individuals with a greater interest in the survey topic are more likely to respond. Respondents in the written group were recruited in two hospitals, potentially indicating greater concern for health problems in general and accessibility in particular. Our results seem to indicate a potential social desirability bias toward the written method against the Internet method, and an avidity bias toward our rural hospital (BMP) against our more urban hospital (CHUS). Although not significant, an avidity bias could explain the greater acceptance in the emergency room.

The use of multiple linear regressions including dummies for survey modes (i.e., Internet = 1 whereas Written = 0; CHUS = 1 whereas BMP = 0; Emergency = 1 whereas Outpatient = 0) and socio-demographic variables allow us to eliminate the selection bias previously noted. Indeed, introduction of these variables in a multiple linear regression allows simultaneous consideration of the impact of other variables and evaluation of the impact of each variable on social acceptance and confidence. The results in Table 5 indicate a negative impact of Internet and CHUS on social acceptance and confidence after controlling for these socio-demographic variables. The Internet questionnaire confirms the social desirability bias existence and potential avidity bias toward the paper mode. However, we are not able to identify which bias dominates, although we suspect that the social desirability bias is stronger. In the case of CHUS, the estimate indicates the existence of an avidity bias toward BMP (i.e., the rural area).

**Table 5:** **Multiple linear regressions**

	**Total sample**		**Hospital sample (model 1)**		**Hospital sample (model 2)**	
	**Acceptance**	**Confidence**	**Acceptance**	**Confidence**	**Acceptance**	**Confidence**
Internet	**−1.103**	**−12.325**				
	(0.008)	(0.000)				
CHUS			**−2.629**	**−13.455**		
			(0.013)	(0.002)		
Emergency					0.537	6.126
					(0.501)	(0.057)
Gender	0.023	**3.632**	0.809	2.699	0.745	2.580
	(0.929)	(0.004)	(0.330)	(0.412)	(0.381)	(0.445)
Age	**0.078**	**0.240**	0.036	0.093	0.043	0.164
	(0.000)	(0.000)	(0.247)	(0.454)	(0.199)	(0.223)
Education	**0.220**	**1.319**	**0.580**	**1.420**	**0.569**	**1.305**
	(0.000)	(0.000)	(0.000)	(0.019)	(0.000)	(0.036)
Income	2.37e-06	2.36e-06	−0.000	−0.000	−0.000	−0.000
	(0.687)	(0.932)	(0.144)	(0.879)	(0.114)	(0.668)
Single	**0.599**	**2.660**	1.035	4.263	0.710	1.935
	(0.029)	(0.039)	(0.224)	(0.205)	(0.410)	(0.568)
Employment	**0.519**	0.232	1.305	2.241	1.382	3.302
	(0.050)	(0.852)	(0.185)	(0.564)	(0.175)	(0.413)
Children	0.570	1.510	1.013	2.631	1.065	3.301
	(0.055)	(0.280)	(0.312)	(0.519)	(0.301)	(0.432)
Constant	**12.604**	**46.842**	**12.125**	**61.617**	**9.489**	**45.387**
	(0.000)	(0.000)	(0.000)	(0.000)	(0.001)	(0.000)
Nb. Obs	1,692	1,688	169	165	165	161
R2	0.074	0.078	0.124	0.098	0.098	0.068

Significant difference in bold at p < 0.05.

The results in Table 5 indicate that education always positively influences social acceptance and confidence in telehealth, which reveals that better educated people are more aware of the benefits of telehealth and more readily accept its development. Age and single status in the total sample also indicate a positive impact on social acceptance and confidence. These variables indicate a seemingly increased interest in health service accessibility with age and single status.

## Discussion

### Summary

Our results indicate the following: 1) selection bias and social desirability bias are potentially less prominent in the Internet survey versus the written survey; 2) scores of social acceptance and confidence in telehealth are higher for treatment than diagnosis and even higher in case of emergency; 3) scores are generally the same when we compare answers given for the respondent and for a member of the family; 4) no difference in scores is found when a health professional, versus a medical doctor, is assisted by telehealth; 5) respondents living in a rural area gave higher scores than the rest of the sample; and 6) the number of years in school is a good predictor of social acceptance and confidence level in telehealth.

### Limitations

Our study presents several limitations that should be corrected in future research. First, the discrepancy between the size of the Internet survey and the written survey may have led to an overrepresentation of certain respondents. Second, the written survey is not randomly selected. Third, the sample identified as a rural area (i.e., BMP) is very small, which could bias the results. Fourth, we cannot control for a yes-saying response bias. Fifth, even if our questionnaire has been validated on a qualitative basis, the survey is not an internationally validated questionnaire and was not tested for internal and external consistency on a quantitative basis. Finally, we should have collected more respondent’s information to better identify the predictors of social acceptance and confidence level in telehealth, particularly with respect to past experiences with telehealth.

### Future works

These limitations suggest the need to conduct new studies on this topic. Indeed, this pilot study did not assess many factors. One topic of interest considers the perception variation related to medical intervention types available to guide stakeholders in developing specific telehealth programs. Additionally, future work should consider environmental factors, ethnicity and fears related to telehealth activities to improve understanding of the main factors influencing social acceptance and confidence level in telehealth. We should also investigate the lack of difference in the scores between real-time remote assistance of a healthcare professional and real-time remote assistance of a physician.

Future works should review the questionnaire validity with the inclusion of a question about the geographical location of the respondent (e.g., postal code) to compare the results from different geographical environments. The results could confirm that rural regions have a better perception of telehealth services than more urbanized regions, such as a region with a university hospital center. Indeed, the CHUS and BMP hospitals showed a significant difference in social acceptability scores and population confidence scores. We think that the BMP population has a better score in both dimensions compared to the CHUS because BMP population is not in proximity to a university hospital with specialized physicians. However, we urge careful interpretation of the results. The number of BMP participants represented only 11.8% of the sample in outpatient clinics, which may have caused an inaccurate estimation due to sampling. Future works are needed to clarify the question of whether a population in a rural region has a better social acceptability and greater confidence in telehealth than a region in proximity to a university hospital. This new information will facilitate better understanding of issues related to telehealth and help telehealth program management developers.

## Conclusions

Our results suggest that the population in Quebec encourages the development of telehealth for real-time diagnosis and distance treatment for regions deprived of healthcare professionals to improve quality of care, especially during emergency situations or among respondents from a rural region.

We obtained a higher number of respondents to the survey by the Internet and this population is more representative of the general socio-demographic attributes of the population of Quebec. In addition, this survey mode potentially generates less social desirability bias because respondents may have answered the questions more fairly and have no incentive to do not give their true opinion [23].

Further studies are needed to improve the understanding of factors influencing social acceptance and population confidence in telehealth.

## Additional files

Additional file 1:
**Questionnaire of social acceptance in telehealth (in French).**
Additional file 2:
**Frequency distribution.**
