# Supplementary material for: Social acceptance and population confidence in telehealth in Quebec
Source: BMC Health Serv Res. 2015 Feb 21;15:72. doi: 10.1186/s12913-015-0727-1 (PMC4338633; doi:10.1186/s12913-015-0727-1)
Supplement: Additional file 1: — Questionnaire of social acceptance in telehealth (in French). [file 12913_2015_727_MOESM1_ESM.doc]

**Additional file 1.**

**Questionnaire of social acceptance in telehealth (in French).**

**Date de naissance (aaaa/mm) : ________/_____**

**Sexe :** Femme  Homme

**Quel est votre degré de scolarité?**

École primaire  École secondaire  DEP  CÉGEP  Baccalauréat

Maîtrise  Ph. D.  Autre

**Quel travail ou profession exercez-vous?**

Temps complet  Temps partiel  Sur appel  Retraité(e)

**Sinon, quelle est votre situation?**

Pas de travail rémunéré  Sans emploi (chômeur)

Étudiant (e)  Congé maladie : Oui  Non

**Quel est votre revenu annuel brut en $ (avant impôt) ?**

< 20 000  20 000 - 34 999  35 000 - 49 999  50 000 - 74 000

>75 000  Refus de répondre

**Quel est votre état matrimonial :**

Célibataire  Marié (e)  Veuf (ve)  Divorcé (e)  Séparé (e)

Conjoint (e) de fait  Refus de répondre

**Avez-vous des enfants?**  Oui  Non

**Nous connaissons au Québec une pénurie de ressources spécialisées en santé. La télésanté est un moyen d’améliorer l’accessibilité aux soins spécialisés dans les régions dépourvues de spécialistes et de rendre les soins plus équitables et à la portée de tous. Pour ce faire, elle permet à des médecins ou professionnels de la santé de communiquer entre eux pour établir, entre autres, un diagnostic ou un plan de traitement.**

**La télésanté consiste à dispenser des soins à distance par biais de technologies de télécommunication audiovisuelle. Ces technologies peuvent offrir un partage d’informations entre médecins ou professionnels de la santé grâce à la transmission d’images, de son ou de tout autres informations en temps réel.**

**Pour les questions suivantes,**

**Indiquer votre degré d’accord et indiquer votre degré de confiance.**

Pour vous-même

Sachant que votre région est dépourvue des ressources médicales requises pour établir le diagnostic de votre maladie ou pour établir un plan de soins pour traiter votre maladie, seriez vous d’accord pour utiliser la télésanté, c’est-à-dire, utiliser un système de caméras vous permettant de consulter à distance un médecin traitant ou un médecin spécialiste localisé dans une autre région?

Totalement  Modérément  Faiblement  Pas du tout

À quel pourcentage se situe votre degré de confiance en la télésanté pour consulter un médecin?

| **%** | **0** | **5** | **10** | **15** | **20** | **25** | **30** | **35** | **40** | **45** | **50** | **55** | **60** | **65** | **70** | **75** | **80** | **85** | **90** | **95** | **100** |
| --- | --- | --- | --- | --- | --- | --- | --- | --- | --- | --- | --- | --- | --- | --- | --- | --- | --- | --- | --- | --- | --- |
|  |  |  |  |  |  |  |  |  |  |  |  |  |  |  |  |  |  |  |  |  |

Pour un membre de votre famille

Sachant que votre région est dépourvue des ressources médicales requises pour établir le diagnostic d’une maladie de l’un de vos proches ou pour établir un plan de soins pour le traiter, seriez vous d’accord pour utiliser la télésanté, c’est-à-dire, utiliser un système de caméras permettant à un membre de votre famille de consulter à distance un médecin traitant ou un médecin spécialiste localisé dans une autre région?

Totalement  Modérément  Faiblement  Pas du tout

À quel pourcentage se situe votre degré de confiance en la télésanté pour consulter un médecin?

| **%** | **0** | **5** | **10** | **15** | **20** | **25** | **30** | **35** | **40** | **45** | **50** | **55** | **60** | **65** | **70** | **75** | **80** | **85** | **90** | **95** | **100** |
| --- | --- | --- | --- | --- | --- | --- | --- | --- | --- | --- | --- | --- | --- | --- | --- | --- | --- | --- | --- | --- | --- |
|  |  |  |  |  |  |  |  |  |  |  |  |  |  |  |  |  |  |  |  |  |

Votre région est dépourvue des ressources médicales requises pour prodiguer des soins spécialisés aux patients de votre région. La télésanté est un moyen d’accéder à ces ressources. Par le biais d’un système de caméras, la télésanté permet la communication en temps réel entre les médecins de votre région et les médecins spécialistes localisés dans d’autres régions.

Vous vous rendez chez votre médecin pour recevoir un traitement spécialisé. Seriez-vous d’accord pour que votre médecin demande à un médecin spécialiste d’une autre région de l’assister en temps réel dans l’exécution des techniques de pointe nécessaires à votre traitement?

Totalement  Modérément  Faiblement  Pas du tout

À quel pourcentage se situe votre degré de confiance en la télésanté pour assister en temps réel un médecin dans l’exécution de techniques de pointe durant votre traitement spécialisé?

| **%** | **0** | **5** | **10** | **15** | **20** | **25** | **30** | **35** | **40** | **45** | **50** | **55** | **60** | **65** | **70** | **75** | **80** | **85** | **90** | **95** | **100** |
| --- | --- | --- | --- | --- | --- | --- | --- | --- | --- | --- | --- | --- | --- | --- | --- | --- | --- | --- | --- | --- | --- |
|  |  |  |  |  |  |  |  |  |  |  |  |  |  |  |  |  |  |  |  |  |

Pour un membre de votre famille

Vous vous rendez chez le médecin d’un membre de votre famille pour qu’il reçoive un traitement spécialisé. Seriez-vous d’accord pour que le médecin demande à un médecin spécialiste d’une autre région de l’assister en temps réel dans l’exécution des techniques de pointe nécessaires à son traitement?

Totalement  Modérément  Faiblement  Pas du tout

À quel pourcentage se situe votre degré de confiance en la télésanté pour assister en temps réel un médecin dans l’exécution de techniques de pointe durant le traitement spécialisé d’un membre de votre famille?

| **%** | **0** | **5** | **10** | **15** | **20** | **25** | **30** | **35** | **40** | **45** | **50** | **55** | **60** | **65** | **70** | **75** | **80** | **85** | **90** | **95** | **100** |
| --- | --- | --- | --- | --- | --- | --- | --- | --- | --- | --- | --- | --- | --- | --- | --- | --- | --- | --- | --- | --- | --- |
|  |  |  |  |  |  |  |  |  |  |  |  |  |  |  |  |  |  |  |  |  |

Votre région est dépourvue des ressources professionnelles requises (infirmières, physiothérapeutes, etc.) pour prodiguer des soins spécialisés aux patients de votre région. La télésanté est un moyen d’accéder à ces ressources spécialisées. Par le biais d’un système de caméras, la télésanté permet la communication en temps réel entre les professionnels de la santé de votre région et ceux localisés dans d’autres régions.

Vous vous rendez à votre clinique médicale ou CLSC pour recevoir un traitement spécialisé. Seriez-vous d’accord pour que le professionnel qui vous traite demande à un spécialiste d’une autre région de l’assister en temps réel dans l’exécution des techniques de pointe nécessaires à votre traitement?

Totalement  Modérément  Faiblement  Pas du tout

À quel pourcentage se situe votre degré de confiance en la télésanté pour assister en temps réel le professionnel de votre région dans l’exécution de techniques de pointe durant votre traitement spécialisé?

| **%** | **0** | **5** | **10** | **15** | **20** | **25** | **30** | **35** | **40** | **45** | **50** | **55** | **60** | **65** | **70** | **75** | **80** | **85** | **90** | **95** | **100** |
| --- | --- | --- | --- | --- | --- | --- | --- | --- | --- | --- | --- | --- | --- | --- | --- | --- | --- | --- | --- | --- | --- |
|  |  |  |  |  |  |  |  |  |  |  |  |  |  |  |  |  |  |  |  |  |

Pour un membre de votre famille

Vous accompagnez l’un des membres de votre famille à la clinique médicale ou CLSC de votre région pour recevoir un traitement spécialisé. Seriez-vous d’accord pour que le professionnel qui traite le membre de votre famille demande à un spécialiste d’une autre région de l’assister en temps réel dans l’exécution des techniques de pointe nécessaires à son traitement?

Totalement  Modérément  Faiblement  Pas du tout

À quel pourcentage se situe votre degré de confiance en la télésanté pour assister en temps réel le professionnel de santé de votre région dans l’exécution de techniques de pointe durant le traitement spécialisé du membre de votre famille?

| **%** | **0** | **5** | **10** | **15** | **20** | **25** | **30** | **35** | **40** | **45** | **50** | **55** | **60** | **65** | **70** | **75** | **80** | **85** | **90** | **95** | **100** |
| --- | --- | --- | --- | --- | --- | --- | --- | --- | --- | --- | --- | --- | --- | --- | --- | --- | --- | --- | --- | --- | --- |
|  |  |  |  |  |  |  |  |  |  |  |  |  |  |  |  |  |  |  |  |  |

Vous vivez dans une région où les médecins travaillant à l’urgence de votre hôpital sont des médecins de famille formés en médecine d’urgence. Ces médecins peuvent être aux prises avec des cas complexes nécessitant l’expertise de spécialistes en traumatologie. Mais votre région est dépourvue de tels spécialistes. La télésanté est un moyen d’accéder à ces médecins spécialistes. Par le biais d’un système de caméras installé dans la salle de traumatologie, les médecins d’urgence de votre région peuvent communiquer en temps réel avec les médecins spécialistes en traumatologie localisés dans d’autres régions.

Vous avez un accident grave et vous êtes transporté à l’urgence de l’hôpital de votre région. Vous êtes inconscient à votre arrivée à l’urgence. Le médecin traitant de l’urgence doit exécuter des techniques d’urgence spécialisées pour vous réanimer ou pour stabiliser votre état de santé. Seriez-vous d’accord pour que le médecin vous traitant à l’urgence demande à un médecin spécialiste en traumatologie d’une autre région de l’assister en temps réel dans l’exécution des techniques d’urgence spécialisées nécessaires à votre réanimation ou à la stabilisation de votre état de santé?

Totalement  Modérément  Faiblement  Pas du tout

À quel pourcentage se situe votre degré de confiance en la télésanté pour assister en temps réel le médecin d’urgence de l’hôpital de votre région dans l’exécution de techniques d’urgence spécialisées nécessaires à votre réanimation ou à la stabilisation de votre état de santé?

| **%** | **0** | **5** | **10** | **15** | **20** | **25** | **30** | **35** | **40** | **45** | **50** | **55** | **60** | **65** | **70** | **75** | **80** | **85** | **90** | **95** | **100** |
| --- | --- | --- | --- | --- | --- | --- | --- | --- | --- | --- | --- | --- | --- | --- | --- | --- | --- | --- | --- | --- | --- |
|  |  |  |  |  |  |  |  |  |  |  |  |  |  |  |  |  |  |  |  |  |

Un membre de votre famille a un accident grave et il est transporté à l’urgence de l’hôpital de votre région. Il est inconscient à son arrivée à l’urgence. Le médecin traitant de l’urgence doit rapidement exécuter des techniques d’urgence spécialisées pour réanimer ou pour stabiliser son état de santé. Vous n’avez pas encore été avisé qu’il est à l’urgence. Seriez-vous d’accord pour que le médecin de l’urgence demande à un médecin spécialiste en traumatologie d’une autre région de l’assister en temps réel dans l’exécution des techniques d’urgence spécialisées nécessaires à sa réanimation ou à la stabilisation de son état de santé, et ce, en votre absence ?

Totalement  Modérément  Faiblement  Pas du tout

À quel pourcentage se situe votre degré de confiance en la télésanté pour assister en temps réel le médecin d’urgence de l’hôpital de votre région dans l’exécution de techniques d’urgence spécialisées nécessaires à la réanimation ou à la stabilisation de l’état de santé d’un membre de votre famille?

| **%** | **0** | **5** | **10** | **15** | **20** | **25** | **30** | **35** | **40** | **45** | **50** | **55** | **60** | **65** | **70** | **75** | **80** | **85** | **90** | **95** | **100** |
| --- | --- | --- | --- | --- | --- | --- | --- | --- | --- | --- | --- | --- | --- | --- | --- | --- | --- | --- | --- | --- | --- |
|  |  |  |  |  |  |  |  |  |  |  |  |  |  |  |  |  |  |  |  |  |

*L’unité d’évaluation des technologies et des modes d’intervention en santé du CHUS vous
remercie de remplir ce questionnaire*
