# Supplementary material for: Social acceptance and population confidence in telehealth in Quebec
Source: BMC Health Serv Res. 2015 Feb 21;15:72. doi: 10.1186/s12913-015-0727-1 (PMC4338633; doi:10.1186/s12913-015-0727-1)
Supplement: Additional file 2: — Frequency distribution. [file 12913_2015_727_MOESM2_ESM.doc]

Additional file 2

Questionnaire items and frequency distribution for the total sample

| Question | Completely # resp (%) | Moderately # resp (%) | Slightly # resp (%) | Not at all # resp (%) | Total # resp (%) | Mean score |
| --- | --- | --- | --- | --- | --- | --- |
| Q1a | 644 (35.64) | 711 (39.35) | 267 (14.78) | 185 (10.24) | 1,807 (100.00) | 2.0 |
| Q1b | 606 (33.54) | 758 (41.95) | 290 (16.05) | 153 (8.47) | 1,807 (100.00) | 2.0 |
| Q2a | 1,024 (57.53) | 553 (31.07) | 140 (7.87) | 63 (3.54) | 1,780 (100.00) | 2.4 |
| Q2b | 966 (54.27) | 595 (33.43) | 153 (8.60) | 66 (3.71) | 1,780 (100.00) | 2.4 |
| Q3a | 968 (54.72) | 583 (32.96) | 155 (8.76) | 63 (3.56) | 1,769 (100.00) | 2.4 |
| Q3b | 932 (52.66) | 596 (33.67) | 179 (10.11) | 63 (3.56) | 1,770 (100.00) | 2.4 |
| Q4a | 1,174 (66.89) | 432 (24.62) | 110 (6.27) | 39 (2.22) | 1,755 (100.00) | 2.6 |
| Q4b | 1,140 (65.03) | 445 (25.39) | 127 (7.24) | 41 (2.34) | 1,753 (100.00) | 2.5 |

Note: # resp indicates the number of response’s choice for a given question (i.e. completely, moderately, slightly or not at all).

Questionnaire items and frequency distribution for the online sample

| Question | Completely # resp (%) | Moderately # resp (%) | Slightly # resp (%) | Not at all # resp (%) | Total # resp (%) | Mean score |
| --- | --- | --- | --- | --- | --- | --- |
| Q1a | 537 (33.84) | 632 (39.82) | 251 (15.82) | 167 (10.52) | 1,587 (100.00) | 2.0 |
| Q1b | 509 (32.07) | 674 (42.47) | 268 (16.89) | 136 (8.57) | 1,587 (100.00) | 2.0 |
| Q2a | 898 (57.53) | 481 (30.81) | 133 (8.52) | 49 (3.14) | 1,561 (100.00) | 2.4 |
| Q2b | 843 (54.00) | 517 (33.12) | 146 (9.35) | 55 (3.52) | 1,561 (100.00) | 2.4 |
| Q3a | 845 (54.48) | 506 (32.62) | 147 (9.48) | 53 (3.42) | 1,551 (100.00) | 2.4 |
| Q3b | 812 (52.35) | 518 (33.40) | 168 (10.83) | 53 (3.42) | 1,551 (100.00) | 2.3 |
| Q4a | 1,020 (66.32) | 379 (24.64) | 104 (6.76) | 35 (2.28) | 1,538 (100.00) | 2.6 |
| Q4b | 989 (64.30) | 395 (25.68) | 117 (7.61) | 37 (2.41) | 1,538 (100.00) | 2.5 |

Note: # resp indicates the number of response’s choice for a given question (i.e. completely, moderately, slightly or not at all).

Questionnaire items and frequency distribution for the written sample

| Question | Completely # resp (%) | Moderately # resp (%) | Slightly # resp (%) | Not at all # resp (%) | Total # resp (%) | Mean score |
| --- | --- | --- | --- | --- | --- | --- |
| Q1a | 107 (48.64) | 79 (35.91) | 16 (7.27) | 18 (8.18) | 220 (100.00) | 2.3 |
| Q1b | 97 (44.09) | 84 (38.18) | 22 (10.00) | 17 (7.73) | 220 (100.00) | 2.2 |
| Q2a | 126 (57.53) | 72 (32.88) | 7 (3.20) | 14 (6.39) | 219 (100.00) | 2.4 |
| Q2b | 123 (56.16) | 78 (35.62) | 7 (3.20) | 11 (5.02) | 219 (100.00) | 2.4 |
| Q3a | 123 (56.42) | 77 (35.32) | 8 (3.67) | 10 (4.59) | 218 (100.00) | 2.4 |
| Q3b | 120 (54.79) | 78 (35.62) | 11 (5.02) | 10 (4.57) | 219 (100.00) | 2.4 |
| Q4a | 154 (70.97) | 53 (24.42) | 6 (2.76) | 4 (1.84) | 217 (100.00) | 2.6 |
| Q4b | 151 (70.23) | 50 (23.26) | 10 (4.65) | 4 (1.86) | 215 (100.00) | 2.6 |

Note: # resp indicates the number of response’s choice for a given question (i.e. completely, moderately, slightly or not at all).
